# Supplementary material for: Non-stem cell lineages as an alternative origin of intestinal tumorigenesis in the context of inflammation
Source: Nat Genet. 2024 Jun 20;56(7):1456–67. doi: 10.1038/s41588-024-01801-y (PMC11250264; doi:10.1038/s41588-024-01801-y)
Supplement: Supplementary file 1 — Reporting Summary [file 41588_2024_1801_MOESM1_ESM.pdf]

nature portfolio

Corresponding author(s): Riccardo Fodde
Last updated by author(s): Apr 30, 2024

Reporting Summary

Nature Portfolio wishes to improve the reproducibility of the work that we publish. This form provides structure for consistency and transparency in reporting. For further information on Nature Portfolio policies, see our Editorial Policies and the Editorial Policy Checklist.

Statistics

For all statistical analyses, confirm that the following items are present in the figure legend, table legend, main text, or Methods section.

n/a	Confirmed	
☐	☒	The exact sample size (*n*) for each experimental group/condition, given as a discrete number and unit of measurement
☐	☒	A statement on whether measurements were taken from distinct samples or whether the same sample was measured repeatedly
☐	☒	The statistical test(s) used AND whether they are one- or two-sided<br>*Only common tests should be described solely by name; describe more complex techniques in the Methods section.*
☐	☒	A description of all covariates tested
☐	☒	A description of any assumptions or corrections, such as tests of normality and adjustment for multiple comparisons
☐	☒	A full description of the statistical parameters including central tendency (e.g. means) or other basic estimates (e.g. regression coefficient) AND variation (e.g. standard deviation) or associated estimates of uncertainty (e.g. confidence intervals)
☐	☒	For null hypothesis testing, the test statistic (e.g. F, t, r) with confidence intervals, effect sizes, degrees of freedom and P value noted<br>*Give P values as exact values whenever suitable.*
☒	☐	For Bayesian analysis, information on the choice of priors and Markov chain Monte Carlo settings
☒	☐	For hierarchical and complex designs, identification of the appropriate level for tests and full reporting of outcomes
☐	☒	Estimates of effect sizes (e.g. Cohen's *d*, Pearson's *r*), indicating how they were calculated

Our web collection on statistics for biologists contains articles on many of the points above.

Software and code

Policy information about availability of computer code

Data collection	Histological analysis: Nanozoomer (Hamamatsu), NDP Viewer (v2, Hamamatsu).<br>Fluorescent images: Confocal LSM 700 (Zeiss), Confocal Stellaris 5 (Leica).<br>Real Time PCRs: 7500 Real Time System (Applied Biosystems).<br>FACS: FACSAria III (BD Biosciences)<br>scRNAseq: Chromium Controller (10X Genomics).<br>sequencing: HiSeq 2500 (Illumina), NovaSeq 6000 (Illumina), DNBSEQ-G400 (MGI).
Data analysis	Statistical analysis was performed in R (v4.2.1) running under Red Hat Enterprise Linux 8.6.<br>For analysis of histology and fluorescent pictures, the following software packages were used: ImageJ (v 2.0.0), QuPath (v 0.4.0), NDPViewer (v 2.9.25)<br>For analysis of FACS data, the following software packages were used: FACSDiva (v.8.0.1, BD Biosciences), FlowJo (V10).<br>Analysis of transcriptomic data was based on publicly available software packages as specified in Methods. Scripts are available upon request.<br>For bulk RNA seq analysis, the following software packages were used: SOAPnuke pipeline (v1), STAR (v 2.7.9a), sambamba (v 0.8.0), subread (v 2.0.3), DESeq2 (v 1.34.0), biomaRt (v 2.52.0), GSVA (v 1.44.5), fgsea (v 1.22.0), ggplot2 (3.4.0), dplyr (v 1.0.10), ComplexHeatmap (v.2.12.1), EnhancedVolcano (v 1.14.0), ISCindex (v 0.0.0.9), survival (v 3.3-1), survminer (v 0.4.9), CiberSortx, MiXCR (v4.6.0), immunarch (v0.9.0).<br>For scRNA seq analysis, the following software packages were used: cellranger (v 7.0.0), Seurat (v 4.1.1), DoubletFinder (v 2.0.3), biomaRt (v 2.52.0), GSVA (v 1.44.5), fgsea (v 1.22.0), ggplot2 (3.4.0), dplyr (v 1.0.10), ComplexHeatmap (v.2.12.1), CytoTRACE (v 0.3.3).<br>For scATACseq analaysis, the following packages were used: Seurat (v 4.1.1), Signac (v 1.9.0), GenomicRanges (v 1.50.2), RIdeogram (v 0.2.2).

For manuscripts utilizing custom algorithms or software that are central to the research but not yet described in published literature, software must be made available to editors and reviewers. We strongly encourage code deposition in a community repository (e.g. GitHub). See the Nature Portfolio guidelines for submitting code & software for further information.

Data

Policy information about availability of data

All manuscripts must include a data availability statement. This statement should provide the following information, where applicable:

- Accession codes, unique identifiers, or web links for publicly available datasets
- A description of any restrictions on data availability
- For clinical datasets or third party data, please ensure that the statement adheres to our policy

All data relevant to this study are made available. Transcriptomic sequencing data has been deposited in the Gene Expression Omnibus (GEO), and is available using the following identifiers: GSE221819 (bulk RNA sequencing of murine tumors); GSE221820 (single cell RNA sequencing of genetically targeted Paneth cells); GSE221818 (bulk RNA sequencing of sorted Paneth cells treated with control and western-style diet). Additional data sets referenced in this study are publicly available in the Synapse and GEO repositories.
Previously published scRNAseq studies were employed relative to the mouse small intestine in homeostasis (Haber et al., GSE92332), upon irradiation (Ayyaz et al., GSE117783), Lgr5 ablation (Singh et al., GSE183299), and upon feeding with western-style diet (Choi et al., scRNAseq: GSE188577, scATACseq: GSE228006). Mouse colonic scRNAseq data from AOM/DSS tumors was obtained from Vega et al. (GSE134255) and bulk RNAseq was from Chen et al. (GSE178145). Human colorectal cancer Bulk RNAseq and scRNAseq studies were retrieved from Guinney et al. (syn2623706), Rajamäki et al. and Lee et al. (KUL cohort: GSE144735; SMC cohort: GSE132465), respectively. Human scRNAseq data from Ulcerative Colitis patients (Smillie et al.) was downloaded from the Broad DUOS platform after a DTA agreement. Single cell ATAC data from human colon was obtained from Becker et al. (GSE201349).

Human research participants

Policy information about studies involving human research participants and Sex and Gender in Research.

Reporting on sex and gender	NA
Population characteristics	NA
Recruitment	NA
Ethics oversight	The study followed the guidelines of the European Network of Research Ethics Committees, in line with European, national, and local regulations. As per national protocols, informed consent was not required for the immune-histological analysis of residual tissue material. Approval for the use of material from IBD patients was obtained from the medical ethical committee of the Erasmus MC under license MEC-2009-041.

Note that full information on the approval of the study protocol must also be provided in the manuscript.

Field-specific reporting

Please select the one below that is the best fit for your research. If you are not sure, read the appropriate sections before making your selection.

☒ Life sciences ☐ Behavioural & social sciences ☐ Ecological, evolutionary & environmental sciences

For a reference copy of the document with all sections, see nature.com/documents/nr-reporting-summary-flat.pdf

Life sciences study design

All studies must disclose on these points even when the disclosure is negative.

Sample size	For the generation of novel mouse models, no statistical test was used to determine the sample size upfront. As these models were evaluated for the first time, there was no data available upfront to predict the needed sample size in a reliably manner. Moreover, the variation in litter size and breeding efficiency of complex genotypes limits the control of experimental group numbers. For in vivo tumorigenesis experiments, at least 10 animals were screened per genotype, except for Lyz1/Apc/Kras/P53 animals due to difficulties in breeding animals with this genotype. RNAseq analysis was done on N = 3 tumors per group, which we considered as statistical minimum given a large effect size. Single cell RNAseq was performed on at least N = 3 animals per genotype, except for Lyz1/Kras and Lyz1/Apc/Kras/P53, that were analyzed for N = 2 animals. Diet scRNAseq data was obtained from N = 3 mice per group, and scATACseq data was obtained from N = 2 mice per group. In vitro organoid reconstitution assays were performed in duplicate on N = 4 mice per group.
Data exclusions	One mouse has been excluded from the scRNAseq analysis, as the number of sorted Yfp cells was too low (below 50 by FACS counts, possibly due to poor Tamoxifen administration) to draw significant conclusions. Occasionally, mice reached their pre-established humane endpoint (e.g. 20% weight loss) after administration of DSS, which led to exclusion of those mice from the study.<br>No human samples have been excluded from the analysis. An exception is the analysis regarding consensus molecular subtypes. Tumors that were classified as 'unknown' (due to non-significant CMS classification) were filtered from the analysis.
Replication	All experiments have been repeated at least N = 3 times, except the scRNAseq analysis of mice with Lyz1-AKP (N = 2) and Lyz1-K genotype (N = 2). Additional validation of results obtained from these samples is provided by corroborative methods, such as flow cytometry analysis, histological analysis and in situ hybridizations. Attempts at replication were successful for the experiments.

Randomization | Mice were randomly assigned to experimental groups after validation of their genotype, and controlling for age and sex.

Blinding | Whenever possible, experimental labels were blinded to the collectors of the data, and labels were added post data collection during the analyses.

Reporting for specific materials, systems and methods

We require information from authors about some types of materials, experimental systems and methods used in many studies. Here, indicate whether each material, system or method listed is relevant to your study. If you are not sure if a list item applies to your research, read the appropriate section before selecting a response.

Materials & experimental systems

n/a	Involved in the study
☐	☒ Antibodies
☒	☐ Eukaryotic cell lines
☒	☐ Palaeontology and archaeology
☐	☒ Animals and other organisms
☒	☐ Clinical data
☒	☐ Dual use research of concern

Methods

n/a	Involved in the study
☒	☐ ChIP-seq
☐	☒ Flow cytometry
☒	☐ MRI-based neuroimaging

Antibodies

Antibodies used

Primary (mouse):
β-catenin (#610154, BD Biosciences)
γ-H2AX (#9718, Cell Signaling)
Gfp (#A-11122, ThermoFisher)
Olfm4 (#D6Y5A, Cell Signaling)
Lyz1 (#A0099, Dako)
Dclk1 (#ab37994, Abcam)
Muc2 (#sc-15334, Santa Cruz)
Chga (#NB120-15160, Novus Bio)
Ecad (#610182, BD Biosciences)
Mki67 (#NB500, Novus Biologicals)
Cd3 (#ab5690, Abcam)
F4/80 (#D2S9R, Cell Signaling)
αSMA (#ab5690, Abcam)

Primary (human)
KI67 (MIB-1 antibody, #790-4286; Ventana)
MUC2 (CCP58, #M7313, Dako)
BEST4 (HPA058564, Sigma-Aldrich)

Fluorescent
Goat anti-Rabbit IgG (H+L) secondary Antibody Alexa Fluor® 488 conjugate (#A32731, Invitrogen)
anti-rabbit Alexa 568 (#A11011, Invitrogen)
anti-mouse Alexa 633 (#A21052, Invitrogen)
phalloidin Alexa 647 (#A22287, Invitrogen)
SiR-actin (#SC001, Spirochrome)
Wheat Germ Agglutinin (#W32466, ThermoFisher Scientific)

FACS
CD31-BV421 (#563356, BD Biosciences)
CD45-BV421 (#563890, BD Biosciences)
TER119-BV421 (#563998, BD Biosciences)
CD24-APC (#1109070, Sony Biotechnology)
cKit-PE (#105808, Biolegend)
cKit-BB515 (#564481, BD Horizon)

FACS (Hashing)
Hashtag 1 Antibody (#155831, Biolegend)
Hashtag 2 Antibody (#155833, Biolegend)
Hashtag 4 Antibody (#155837, Biolegend)
Hashtag 7 Antibody (#155843, Biolegend)
Hashtag 10 Antibody (#155849, Biolegend)

Validation | All antibodies used in this study has been validated by the providers by western blot analysis and stainings on cell-lines/tissues known to produce the particular protein. Antibodies were titrated to optimize the stainings on sections of the mouse small intestine.

Animals and other research organisms

Policy information about studies involving animals; ARRIVE guidelines recommended for reporting animal research, and Sex and Gender in Research

Laboratory animals	For all experiments, C57BL/6 mice were randomly assigned to experimental groups after matching for gender, age of 8-12 weeks, and genotype. All protocols involving animals were approved by the Dutch Animal Experimental Committee and in accordance with the Code of Practice for Animal Experiments in Cancer Research established by the Netherlands Inspectorate for Health Protections, Commodities and Veterinary Public Health. Animals were bred and maintained in the Erasmus MC animal facility (EDC) under conventional specific pathogen-free conditions. Dark/light cycles were maintained between 7PM-7AM (dark) and 7AM-7PM (light); humidity was kept around 50% (with min 45%, max 65%) and temperature between 20-24°C.<br>The following strains were used and bred with different combinations:<br>Lgr5CreERT2-EGFP (#008875, Jackson Lab)<br>pLysCreERT2 (kind gift from H. Clevers Lab)<br>R26LSL-tdTomato (#007908, Jackson Lab)<br>R26LSL-YFP (#006148, Jackson Lab)<br>Apc15lox (#029275, Jackson Lab)<br>KrasLSL-G12D (#008179, Jackson Lab)<br>Tp53flox (#008462, Jackson Lab)<br>Lgr5DTR-EGFP (MGI:5294798, kind gift from F. de Sauvage Lab)<br>c-KitCreERT2 (MGI:5543260, kind gift from D. Saur Lab)
Wild animals	The study did not involve wild animals
Reporting on sex	All experiments have been performed with male and female animals. Wherever possible, mice were assigned to experimental groups to ensure equal distribution of sexes. Characteristics of the mice including sex will be added to the Figure source data.
Field-collected samples	The study did not involve samples collected from the field
Ethics oversight	All protocols involving animals were approved by the Dutch Animal Experimental Committee and in accordance with the Code of Practice for Animal Experiments in Cancer Research established by the Netherlands Inspectorate for Health Protections, Commodities and Veterinary Public Health.

Note that full information on the approval of the study protocol must also be provided in the manuscript.

Flow Cytometry

Plots

Confirm that:

☒ The axis labels state the marker and fluorochrome used (e.g. CD4-FITC).

☒ The axis scales are clearly visible. Include numbers along axes only for bottom left plot of group (a 'group' is an analysis of identical markers).

☒ All plots are contour plots with outliers or pseudocolor plots.

☒ A numerical value for number of cells or percentage (with statistics) is provided.

Methodology

Sample preparation	Crypts were purified from the small intestine, and enzymatically dissociated with TryplE to obtain a single cell suspension. Cells were stained with fluorescent antibodies (Cd24, cKit, Cd31, Cd45, Ter119) and with Hashing antibodies. Paneth cells were enriched by Fluorescence-activated cell sorting (FACS) according to the SSChiCD24hicKithi gate or by the presence of Yfp/td-Tomato signal
Instrument	FACSAriaIII (BD Biosciences)
Software	Raw data has been processed with FACSDiva™, and the gating strategy was visualized with FlowJo™ v10.
Cell population abundance	Crypt-purified single cell preparations consisted of 2-6% Paneth cells, defined by SSC/Cd24/cKit (see Suppl. Fig. 4b). The purity of post-sorted cells was evaluated by re-analysis with FACS (% of sorted cells mapping in same gate). This purity assessment was in the range of 97-99% (for detailed protocol, see Schewe et al. 2017, JOVE).<br>Viability post sorting, reanalyzed by FACS using DAPI at different time points post sorting till 2 hours (on ice), was > 80-95% for Paneth cells.
Gating strategy	The gating strategy is visualized in Suppl. Fig. 3. In brief, live epithelial cells were obtained by filtering out debris, dead cells and doublets (top panels), Subsequently, Paneth cells were identified based on high SSC-A and high Cd24. This gate was further purified based on high cKit levels. Traced cells were sorted based on high levels of either Yfp or td-Tomato.

☒ Tick this box to confirm that a figure exemplifying the gating strategy is provided in the Supplementary Information.
